# Longitudinal fluctuations in PD1 and PD-L1 expression in association with changes in anti-viral immune response in chronic hepatitis B

**DOI:** 10.1186/1471-230X-12-109

**Published:** 2012-08-16

**Authors:** Zhang Wenjin, Peng Chuanhui, Wan Yunle, Shaikh Abdul Lateef, Zheng Shusen

**Affiliations:** 1 Key Laboratory of Combined Multi-organ Transplantation, Ministry of Public Health, First Affiliated Hospital, School of Medicine, Zhejiang University, 79 Qingchun Road, Hangzhou, 310003, China; 2 Department of Hepatobiliary Surgery, Sun Yat-Sen Memorial Hospital, Sun Yat-Sen University, Guangzhou, 510120, Guangdong Province, China

**Keywords:** PD1, PD-L1, Hepatitis B

## Abstract

**Background:**

Controversy exists regarding the role of PD1 and its ligand PD-L1 in chronic hepatitis B infection. In some studies, persistent HBV infection has been attributed to high levels of PD-1 and PD-L1 expression on HBV-specific T-cells and antigen-presenting cells (APCs) respectively. Other studies revealed that the up-regulation of PD-1 and PD-L1 during an acute inflammation phase is required to offset increasing positive co-stimulatory signals to avoid severe damage by an over-vigorous immune response.

**Methods:**

Fifteen chronic hepatitis B patients, with inflammatory flare episode, were recruited prospectively. Based on serum HBV-DNA, HBsAg load, and ALT values, inflammatory flare episode were divided into initial, climax, decline and regression phase. Blood sample and liver biopsy tissues from each individual were taken in these 4 phases respectively. Circulating and intra-hepatic PD1 and PD-L1 expression levels were monitored throughout the inflammatory flare episode by flow cytometry and immunostaining and these expression levels were related to the HBV-specific T-cell changes, expression of pro-inflammatory cytokines, HBV-DNA replication and HBV antigen load.

**Results:**

]The levels of PD-1 and PD-L1 expressions were significantly up-regulated in the inflammation ascending phase, initial and climax period and in parallel with HBV-specific colon expansion. It showed increasing the level of serum ALT and decreasing the HBV-DNA loads. As the level of inflammation reduced, the circulating and intra-hepatic PD1 and circulating PD-L1 decreased progressively in concordance with serum ALT, HBV-DNA and HBsAg loads decreased except intra-hepatic PD-1 expression. Intra-hepatic PD-L1 expression did not decrease significantly during the regression phase of inflammation compared to that in prior period. The intra-hepatic PD-L1 expression remained relatively on higher level when serum HBV-DNA load and ALT decreased to approximately normal range.

**Conclusion:**

The relatively high level of intra-hepatic PD-L1 expression during the inflammatory regression period may contribute to constitute an immunosuppressive microenvironment, which facilitate persistent HBV infection via the inhibition of HBV-specific T cell clonal expansion.

## Background

Hepatitis B virus (HBV) infection results in the heterogeneous diseases states that vary greatly in HBV replication, liver pathology damage and outcome from person to person. Some patients control infection efficiently and are able to eliminate the virus either without developing evident liver inflammation or a self-limited acute hepatitis syndrome that resolves without long-term periodic inflammatory episodes. Other patients are unable to clear the virus and develop persistent HBV infection.

Anecdotal evidence from clinical observation has shown that the chronic hepatitis B infection often manifests clinically with repeated inflammatory episodes marked by fluctuating levels of serum ALT and HBV-DNA. Levels of serum HBV-DNA and HBV antigens load progressively decrease throughout the inflammatory flare. During immune inactive period, HBV antigens begin to re-accumulated until they reach a high enough level to trigger another round of inflammation.

In recent studies, the inability to eliminate virus in patients with chronic hepatitis B has been attributed to high levels of expression of programmed death 1 (PD-1) and its ligand (PD-L1/B7-H1) on viral antigen-specific T-cells and antigen -presenting cells (APCs) respectively[1-9]. The interaction between this pair of co-inhibitory molecules has been shown to contribute directly to T-cell dysfunction and poor control of viral replication. Blocking the PD-1/PD-L1 interaction in vitro reversed exhausted cytokine production and proliferation of these HBV-specific T cells [1,4-6]. Thus, up-regulation of PD1 and PD-L1 are generally considered markers of malfunction of the HBV-specific immune response.

In contrast, other studies revealed that negative co-stimulatory molecules play a key role in maintenance of homeostasis [10,11]. Studies showed that delayed up regulation of PD1 and PD-L1 were attributed to fulminant liver inflammation in patients with acute hepatitis B and finally led to acute liver failure [12-14]. Previous studies revealed that in patients with acute hepatitis B, PD-L1 expression levels were up regulated during the ascending phase of inflammation and decreased when inflammation regressed. This phenomenon suggested that a balance exists between the negative and positive co-stimulatory molecules on activated immune cells.

In order to elucidate the role of PD1 and PD-L1 in the hepatitis B immune response, the relationship between the PD1/ PD-L1 expression and immune response events, such as HBV-specific CD8 T-cell expansion and contraction, pro-inflammatory cytokines expression, viral load, and disease pathogenesis should be analyzed.

The majority of previous studies of the PD1 and PD-L1 expression in chronic hepatitis B patients were retrospective and cross-sectional. In this study, patients were enrolled prospectively and inflammatory flare period were divided into initial, climax, decline and regression phase. This study is novelty in its prospective and longitudinal analysis of PD1 and PD-L1 expression in chronic hepatitis B patients throughout inflammatory flare period.

The results showed that PD-1/PD-L1 expression was significantly up-regulated during the inflammatory climax period. During the regression period, when serum ALT decrease to approximately normal levels, intra-hepatic PD-L1 expression on Kupffer cells did not decrease significantly compared to that in prior phase. These data suggest that the relatively high level of intra-hepatic PD-L1 expression during the inflammatory regression period may contribute to constitute an immunosuppressive microenvironment. The immunosuppressive microenvironment in liver may facilitate persistent HBV infection via the transmission of co-inhibitory signals to activated T-cells, and finally lead to the next round of liver inflammation.

## Methods

### Subjects

Chronic hepatitis B patients were followed-up in the out-patient clinic by detection of serum ALT, HBV-DNA and HBs-Ag load. Subjects who developed hepatitis B flare episode during follow up period were enrolled in this study. Serum HBV-DNA load more than 5 × 10^3^ copies/mL , and serum ALT level near to 5 times the upper limit of normal, were considered as the onset of initiation of inflammation. HBV tolerance patients and fulminant hepatitis B patients were excluded from this study. The recruiting work was stopped when the fifteenth patient was enrolled. Each of these fifteen patients was followed with protocol visit for a median period of 4 months (range, 3–5 months). Liver biopsy was performed according to ultrasound guide in appointed time point as mentioned following:

Written informed consent was obtained from each enrolled patient, and the study protocol was approved by the Ethics Committee of our unit. Reports of all patients were negative for anti-hepatitis D virus antibodies, anti-hepatitis C virus (anti-HCV) antibodies, anti-HIV1,2 antibodies, and auto-antibodies. The clinical data of these enrolled patients at the first time point (initial phase of inflammation) are summarized in Table 1.

**Table 1:** Patients demographic data and levels of serum markers at time of initial presentation

**Patient no.**	**Gender**	**Level of serum ALT ( IU/L)**	**Level of serum HBsAg (ng/mL)**	**HBV-DNA (copies/mL)**	**Expression of HLA-A**_**2**_
1	male	200	527	1 × 10^7^	positive
2	male	120	513	1.5 × 10^7^	positive
3	male	135	468	6.3 × 10^6^	positive
4	female	122	491	6.1 × 10^6^	positive
5	male	150	132	8.7 × 10^6^	positive
6	female	132	401	1.3 × 10^5^	positive
7	female	197	401	5.1 × 10^6^	positive
8	male	200	441	1.1 × 10^5^	positive
9	male	101	356	7.5 × 10^4^	positive
10	female	157	437	3.3 × 10^6^	positive
11	male	156	491	2.0 × 10^5^	positive
12	female	189	411	7.0 × 10^5^	positive
13	female	172	328	5.4 × 10^6^	positive
14	male	194	396	1.9 × 10^6^	positive
15	female	200	567	1.1 × 10^7^	positive

Abbreviations: *ALT*, alanine transaminase; *HBsAg*, hepatitis B surface antigen; *HBV*, hepatitis B virus; *HLA,* human leukocyte antigen.

Heparinized blood samples used for the separation of peripheral blood mononuclear cells (PBMCs) and liver biopsy tissue specimens were obtained from patients at 4 time points:

**Time 1** (T1) - Initial inflammation phase: High HBV-DNA load, a high HBsAg load, and serum ALT values were no more than 5 times the upper limit of normal (normal range: 10–40 IU/L).

**Time 2** (T2) - Climax phase of inflammation: Reduced viremia and ALT levels elevated to greater than 10 times the upper limit of normal (normal range: 10–40 IU/L).

**Time 3** (T3) - Decline phase of inflammation: Serum ALT levels decreased by greater than 50% of values in the T2 phase.

**Time 4** (T4) - Regression phase of inflammation: Serum HBV-DNA decreased to the level less than 5 × 10^3^ copies/mL and serum ALT level less than 100 IU/L.

### Hepatitis serology

HBsAg, anti-HBs, anti-HBc, HBeAg,anti-HBe, anti-HDV, anti-HCV, and anti–HIV titers were measured using an enzyme immunoassay kit. Serum HBV-DNA was quantified using a sensitive real-time polymerase chain reaction (RT-PCR) technique.

### Pentamers and PD1 analysis

The following antibodies were used for PBMC staining: anti-CD8-FITC, anti-PD1-APC and anti-HLA-A2 (Bio Legend, San Diego, CA USA). HBV-specific CD8+ T-cells were identified with human leukocyte antigen A2 (HLA-A2) pentamers containing core 18–27 peptide (FLPSDFFPSV), evn 335–343 peptide (FLLTRILTI) and pol 575–583 peptide (FLLSLGIHL), which were labeled with the fluorochrome PE.

PBMCs from peripheral blood were incubated for 15 min at 37°C with 1 μg of PE-labeled pentamer complex in PBS and then washed twice. Cells were then incubated with saturating concentrations of directly conjugated anti-CD8–FITC and PD1-APC mAb, for 15 min. After washing the cells twice, they were analyzed immediately on a FACSCalibur flow cytometer using Cell Quest software (BD biosciences, East Rutherford NJUSA).

Lymphocytes were gated according to their physical parameters. All patients were HLA-A2 positive.

Pentamer-positive responses are reported as the percentage of pentamer-positive T-cells among the total CD8 population. Frequencies of pentamer-positive cells in healthy control were not exceeding 0.08% of total circulating CD8 cells. The expansion (n-fold) was calculated as the ratio of the percentage of pentamer-positive T cells between the T1 and T2 periods.

### B7H1 expression analysis

B7-H1 expression on circulating myeloid dendritic cells (mDCs) was measured using 1 mL of fresh heparinized peripheral blood. Cells were lysed with FACS lysing solution (BD biosciences, East Rutherford NJUSA) to remove RBCs and then incubated with antibodies against B7-H1-PE and CD11c-PEcy7 for 20 min at room temperature. After washing twice with PBS, the cells were analyzed by flow cytometry on a FACSCalibur flow cytometer (BD Biosciences).

### Immunohistochemical staining

Acetone-fixed liver tissue cryosections (5 μm) were incubated with anti–PD-1, anti–PD-L1 and anti-CD8 (Abcam plc, Cambridge, UK) antibodies overnight at 4°C after blocking endogenous peroxidase activity with 0.3% H_2_O_2_. Positive cells (brown color) were counted in high power fields (hpf) by 2 independent observers. PD-1 and PD-L1 immunofluorescence dual staining was performed. In brief, liver tissues were incubated overnight with primary antibodies at 4°C followed by secondary antibodies for 45 minutes at room temperature. Anti- PD-1 and PD-L1 (both diluted 1:50; Santa Cruz Biotechnology, Santa Cruz, CA USA) and anti- CD8, CD68, CD31, CD11c and (all diluted 1:50; Dako, Glostrup, Denmark) were used as primary antibodies. Fluorescein isothiocyanate– conjugated immunoglobulin G and fluorochrome phycoerythrin -conjugated immunoglobulin G (Santa Cruz Biotechnology) were used as secondary antibodies.

6. For RT-PCR, total RNA from liver biopsy tissue as indicated was isolated using RNeasy kits (Qiagen, Venlo, Netherlands). cDNA synthesis was performed using SuperScript One-Cycle cDNA kit (Life Technologies, Carlsbad, CA USA). The cDNA served as a template for real-time PCR using Fast SYBR Green Master kit (Life Technologies). The following primers for RT-PCR were used: human IFNγ primers, 5 -GCATCCAAAAGAGTGTGGAG-3' (forward) and 5-GCAGGCAGGACAACCATTAC-3'(reverse); human TNFα primers 5- GAGTGACAAGCCTGTAGCC-3 (forward) and 5-GAGGACCTGGGAGTAGATGA-3 (reverse); and human glyceraldehyde-3-phosphate dehydrogenase (GAPDH) primers, 5 -AACAGCGACACCCACTCCTC −3(forward) and 5 -GGAGGGGAGATTCAGTGTGGT −3(reverse). All reactions were performed in triplicate. IFNγ and TNFα mRNA expression of different group specimens were normalized to glyceraldehydes 3-phosphate dehydrogenase (GAPDH). Relative mRNA levels are presented as unit values of 2^-ΔΔCt^, where Ct is the threshold cycle value defined as the fractional cycle number at which the target fluorescent signal passes a fixed threshold above baseline.

### Statistical analysis

The experimental data were analyzed using SPSS 13.0 software (IBM, Amonk, NY USA). Quantitative data are described using mean ± standard deviation (SD). Comparisons between groups were analyzed using variance or nonparametric tests (Kruskal-Wallis and Mann–Whitney U tests). p < 0.05 was considered significant.

## Results

### Clinical outcome

At baseline, all enrolled patients had high serum viral and HBsAg loads. The highest HBV viral load was greater than 1.5 × 10^7^ (copies/mL). The lowest HBV viral load was greater than 1 × 10^5^ (copies/mL). The mean serum HBsAg load of 15 patients was 433.6 ± 115.4 (ng/ml). During the Climax phase of inflammation (T2), a significant increase in serum ALT levels was paralleled by a profound decrease in the serum HBV load in all patients. Ten of 15 patients showed a low level of HBV-DNA replication from 5 × 10^3^ to 1.5 × 10^4^ copies/mL by the T4 period while five patients had serum HBV-DNA less than 5 × 10^3^ copies/mL.

### Longitudinal analysis of HBV-specific pentamer from T1 to T4

**Figure 1:**
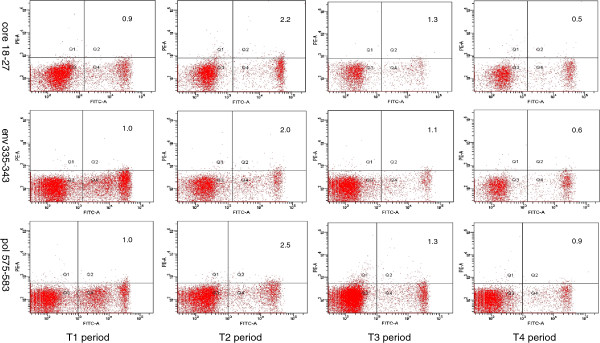
**Representative dot plots of pentamer staining for patients in T1, T2, T3 and T4 sequence.** Fresh heparinized peripheral blood samples were lysed with FACS lysing solution and then incubated with antibodies against pentamers and CD8-FITC for 20 min at 4°C. The numbers in the upper right quadrants indicate the frequency of pentamers in total circulating CD8+ cells.

**Figure 2:**
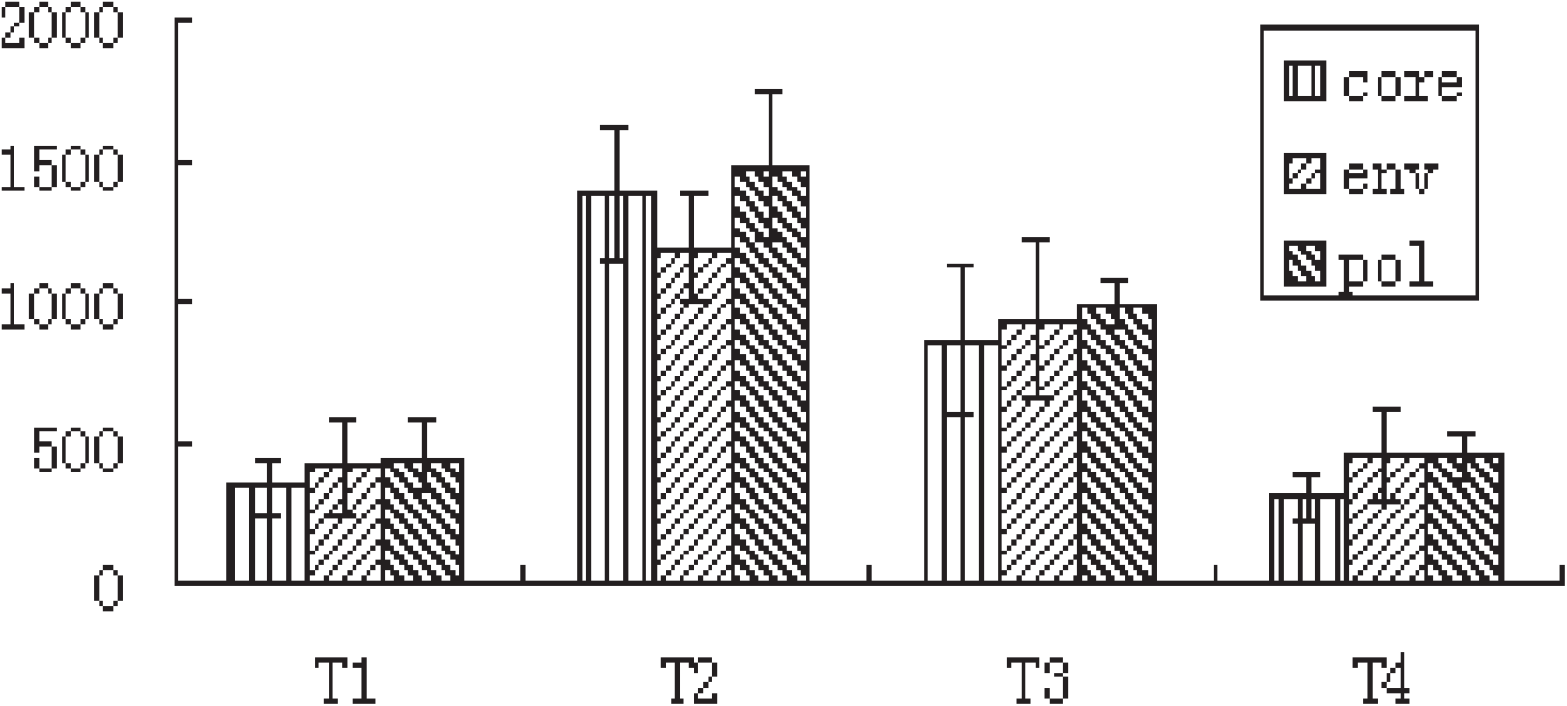
**The numbers of core-, env- and pol-PE positive cells in 50000 CD8+ cells were compared betweemT1,T2,T3 and T4.** The number of core, env and pol increased significantly from T1 to T2, and decreased progressively from T2 to T4. core, env and pol, n = 15, P _T1 vs T2_ <0.01, P _T2 vs T3_ <0.05, P _T3 vs T4_ < 0.01.

**Figure 3:**
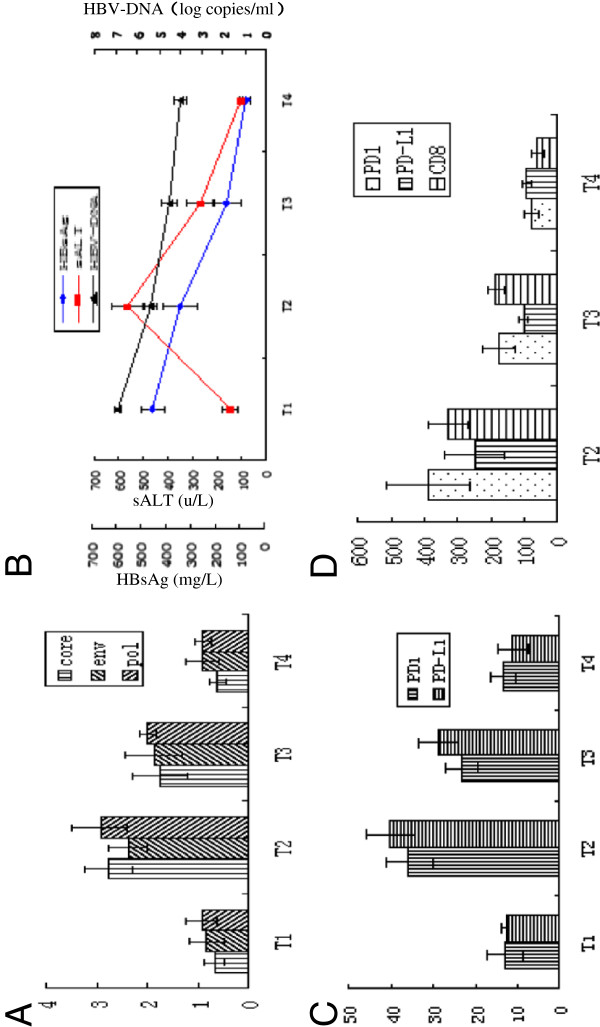
**Correlation between PD1, PD-L1 expression and serum pentamer frequency, ALT level, HBsAg and HBV-DNA load.** (**A**) Percentage of core, even and pol from T1 to T4. The frequency of pentamers including core 18–27, even 335–343 and pol 575–583 increased upon initial of inflammation (T1), reached peak level during climax of inflammation (T2) and decreased gradually during regression of inflammation (T4). (core, even and pol, n = 15, P _T1 vs T2_ <0.01, P _T2 vs T3_ <0.05, P _T3 vs T4_ < 0.01) (**B**) Fluctuation of sALT, HBsAg and HBV-DNA from T1 to T4. From T1 to T2, serum ALT level increased significantly, in parallel with pentamer cells clonal expansion, and decreased significantly in concordance with pentamer cells clonal contraction from T3 to T4. ( sALT, n = 15, P _T1 vs T2_ <0.01, P _T2 vs T3_ <0.05, P _T3 vs T4_ <0.05) Serum HBsAg and HBV-DNA load decreased during inflammation flare up period. (HBsAg and HBV-DNA copies, n = 15, P _T1 vs T2_ <0.01, P _T2 vs T3_ <0.01, P _T3 vs T4_ < 0.01) (**C**) Percentage of circulating PD1 and PD-L1 from T1 to T4. From T1 to T2 period, in parallel with pentamer cells clonal expansion, circulating PD1 and PD-L1 expression increased significantly. From T3 to T4 period, parallel with pentamer cells clonal contraction, circulating PD1 and PD-L1 expression decreased significantly. (P _T1__vs__T2_ <0.01, P _T2 vs T3_ <0.05, P _T3 vs T4_ < 0.01 n = 5) (**D**) The number of intra hepatic CD8, PD1 and PD-L1 positive cells from T2 to T4. DuringT2 period, the number of intra-hepatic PD1, PD-L1 and CD8 reached the highest level and they were decreased significantly in T3 period. From T3 to T4 period, number of intra-hepatic PD-1 and CD8 positive cells further decreased significantly, however PD-L1 did not. (PD1,PD-L1 and CD8, P_T2 vs T3_ < 0.01; PD1 and CD8, P _T3 vs T4_ < 0.01; PD-L1, P _T3 vs T4_ > 0.05; n = 5).

Multiple HBV-specific T cell responses were detected throughout the clinical course from the T1 to the T4 period. During the T1 period, the levels of core18-27, env335-343 and pol575-583 were 0.69% ± 0.29%, 0.84% ± 0.33% and 0.94% ± 0.30% respectively; significantly higher than the healthy control level (which never exceeded 0.08%). During the T2 period, the frequency of core18-27, env335-343 and pol575-583 further increased significantly in all patients. Core 18–27 was detectable at a frequency of 2.77% ± 0.45% of total circulating CD8+ cells (1300 core18-27 cells in 50,000 CD8+ cells), reaching a frequency of 3.0% in 2 patients. The levels of env335-343 and pol575-583 in CD8+ cells also increased significantly, reaching the frequencies of 2.38% ± 0.37% (1200 env 335–343 cells in 50,000 CD8+ cells) and 2.93% ± 0.54% (1460 pol 575–583 cells in 50,000 CD8+ cells) respectively. During the decline phase of inflammation (T3), the frequency of core18-27, env335-343 and pol575-583 in total circulating CD8+ cells were decreased from 2.77% ± 0.45% to 1.75% ± 0.55%, from 2.38% ± 0.37% to 1.88% ± 0.55% and from 2.93% ± 0.54% to 1.99% ± 0.16% respectively. During the regression phase of inflammation (T4), the frequency of these 3 pentamers further decreased to no more than 1% of total circulating CD8+ cells (Figure 1, Figure 2, Figure 3A).

### Longitudinal analysis of PD1 and PD-L1 expression from T1 to T4 in patients with chronic hepatitis B

**Figure 4:**
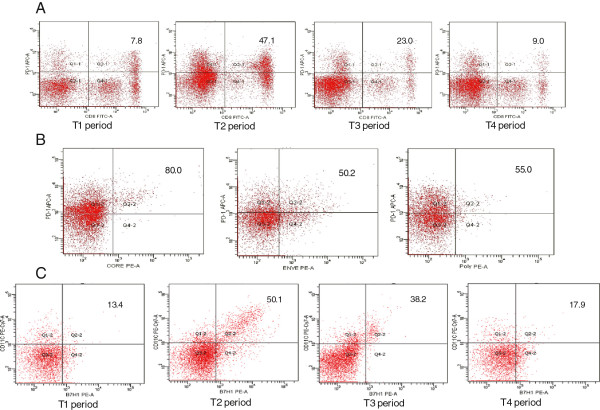
**(A) Representative dot plots of PD1 and CD8 double staining for patients in T1,T2,T3 and T4 sequence.** Fresh heparinized peripheral blood samples were lysed with FACS lysing solution and then incubated with antibodies against PD1-APC and CD8-FITC for 20 min at 4°C. The numbers in the upper right quadrants indicate the frequency of PD1 in total circulating CD8+ cells. (**B**) Representative dot plots of PD1 and pentamer double staining for patients in T2 period. Fresh heparinized peripheral blood samples were lysed with FACS lysing solution and then incubated with antibodies against pentamer-PE and PD1-APC for 20 min at 4°C. The numbers in the upper right quadrants indicate the frequency of PD1-APC in circulating core18-27, enve335-343 and pol575-583 cells. (**C**) Representative dot plots of PD-L1 and CD11c double staining for patients in T1, T2, T3 and T4 sequence. Fresh heparinized peripheral blood samples were lysed with FACS lysing solution to remove RBCs and then incubated with antibodies against PD-L1-PE and CD11c-PEcy7 for 20 min at 4°C. The numbers in the upper right quadrants indicate the frequency of PD-L1 and CD11c dual positive cells.

**Figure 5:**
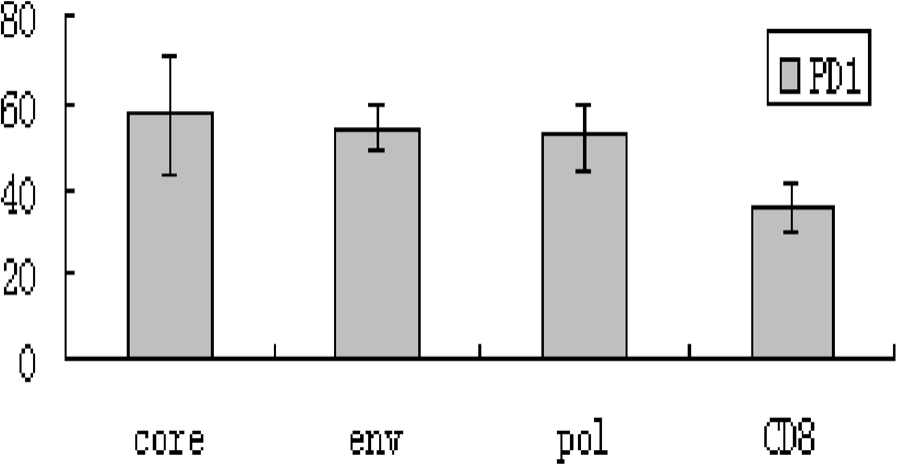
**The frequency of PD1 expression on pentamers in T2 compared with which expressed on circulating CD8.** During T2 period, the frequency of PD1 expression on pentamers were higher significantly than that of expressed on total circulating CD8 ( P core vs CD8 <0.05, Penv vs CD8 <0.05, P pol vs CD8 < 0.05 ).

The frequency of PD1 and PD-L1 expression on circulating CD8+ T cells and mDCs respectively were detected from the T1 to T4 periods. From T1 to T2, the proportion of PD1 positive cells increased significantly from 13.1% ± 4.2% to 35.7% ± 5.8% of total circulating CD8+ cells. During the T3 period, the proportion of PD1-positive cells decreased significantly from 35.7% ± 5.8% in the T2 period to 23.2% ± 4.05% of total circulating CD8+ T cells. During the T4 period, the proportion of PD1-positive cells further decreased to 13.4% ± 3.0%, which approaches the levels found in the T1 period (Figure 4A, Figure 3C). The frequency of PD1 expression on pentamers was significantly higher than that among total circulating CD8+ T cells. During T2 period the frequency of PD1 expression on pentamers of core18-27, env335-343 and pol575-583 were 57.5% ± 13.6%, 54.2% ± 5.6% and 53.3% ± 7.5% respectively (Figure 4B, Figure 5).

The dynamic PD-L1 expression was also detected on circulating CD11c + mDCs from the T1 to the T4 period. The level of PD-L1 expression on CD11c + cells increased significantly after the onset of liver inflammation. The highest frequencies of PD-L1and CD11c dual positive cells were found during the T2 period. Following T2, the frequency of PD-L1and CD11c dual-positive cells decreased progressively from 40.1% ± 5.82% in T2 to28.86 ± 4.95% in T3 period. During T4, PD-L1 expression levels further decreased to11.2% ± 3.54%, which was lower than the levels detected in the T1 period (Figure 4C, Figure 3C).

### Longitudinal analysis of intrahepatic CD8, PD-1and PD-L1 expression from T2 to T4

**Figure 6:**
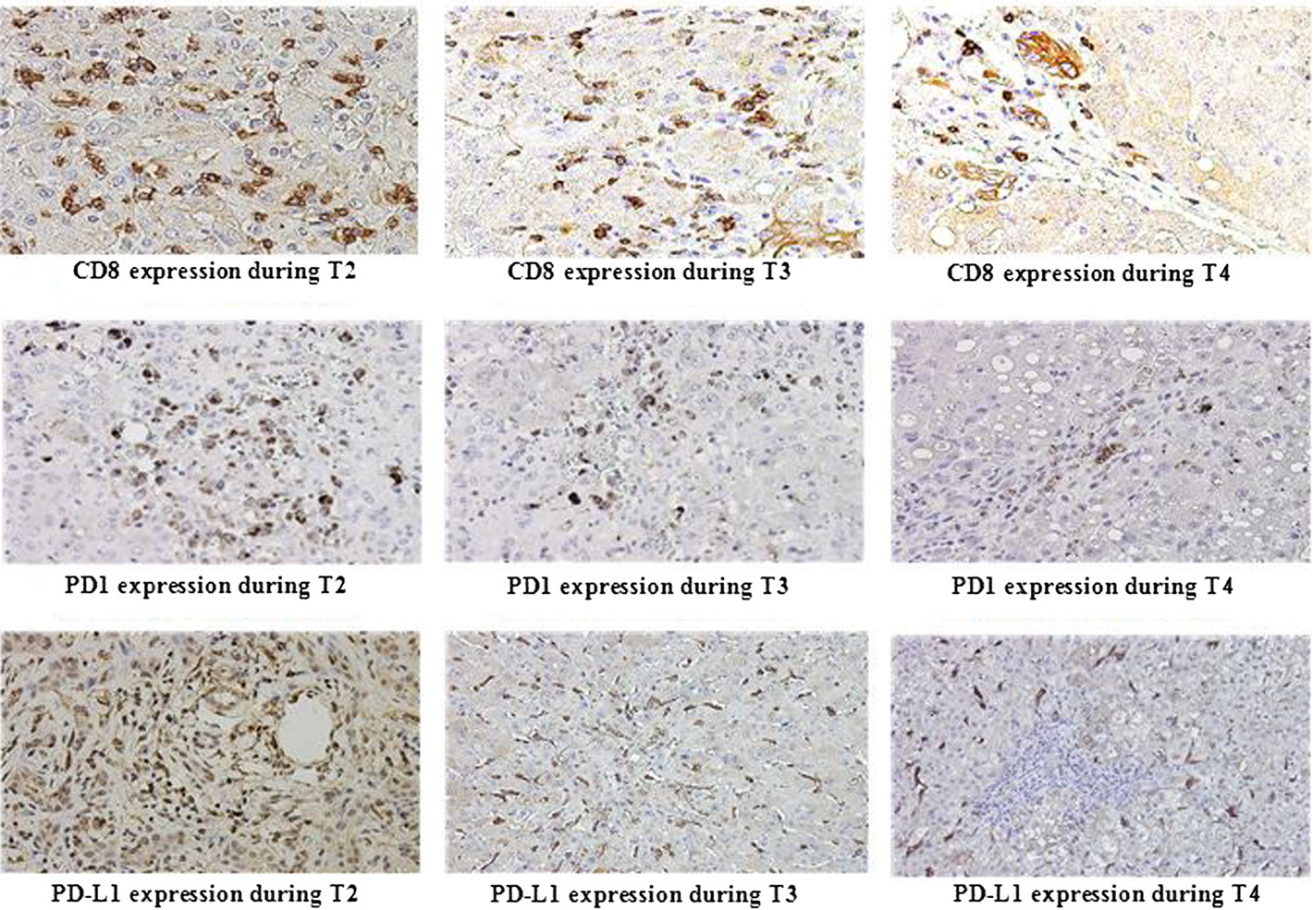
**Immunohistochemical staining for intrahepatic CD8-positive, PD-1-positive and PD-L1-positive cells in patients with chronic hepatitis B.** Representative figures for patients in T2, T3 and T4 sequence. n = 5, High-power field, original magnification 400×. During T2 period, CD8-positive, PD-1-positive or PD-L1-positive cells infiltrated extensively in liver tissue and they were decreased significantly in T3. In T4, the CD8-positive and PD-1-positive cells were less observed in lobular areas, while PD-L1 positive cells were also frequently observed in liver lobular region.

Serially sectioned liver biopsy specimens of enrolled patients from periods T2 to T4 period were examined for CD8, PD1 and PD-L1 expression by immunohistochemistry. During the T2 period, CD8+ positive cells infiltrated the portal tract and extend into the liver parenchyma. The number of infiltrating CD8+ positive cells decreased from 328 ± 58/ hpf in T2 to 186 ± 21/hpf in T3 period. During regression phase of inflammation (T4), several CD8+ positive cells were observed in liver lobular area, most aggregated in fibrous septa (Figure 6, Figure 3D).

Using serial section, intra-hepatic PD1 and PD-L1 expression was also detected dynamically by immunohistology. During the T2 period, PD1 and PD-L1 positive cells were abundantly observed in periportal and lobular areas of the liver. During the T3 period, the numbers of PD1 and PD-L1 positive cells in the liver lobe area decreased significantly. The mean number of infiltrating PD1 positive cells decreased from 388 ± 122/ hpf in T2 to 174 ± 53/ hpf in T3. The number of infiltrating PD-L1-positive cells also decreased from 250 ± 92 / hpf in T2 to 103 ± 14 in T3. In T4, PD1 positive cells were almost absent in the lobular region, with most observed in fibrous septa and the portal tract, while PD-L1-positive cells were also frequently observed in the lobular region of the liver. The number of PD-L1 positive cells decreased from 103 ± 14/hpf in T3 to92 ± 13/hpf in T4. There was no significant difference between the number of PD-L1-positive cells in T3 and T4 (Figure 6, Figure 3D).

### Co-localization of intra-hepatic PD-L1 with various cell markers in T4

**Figure 7:**
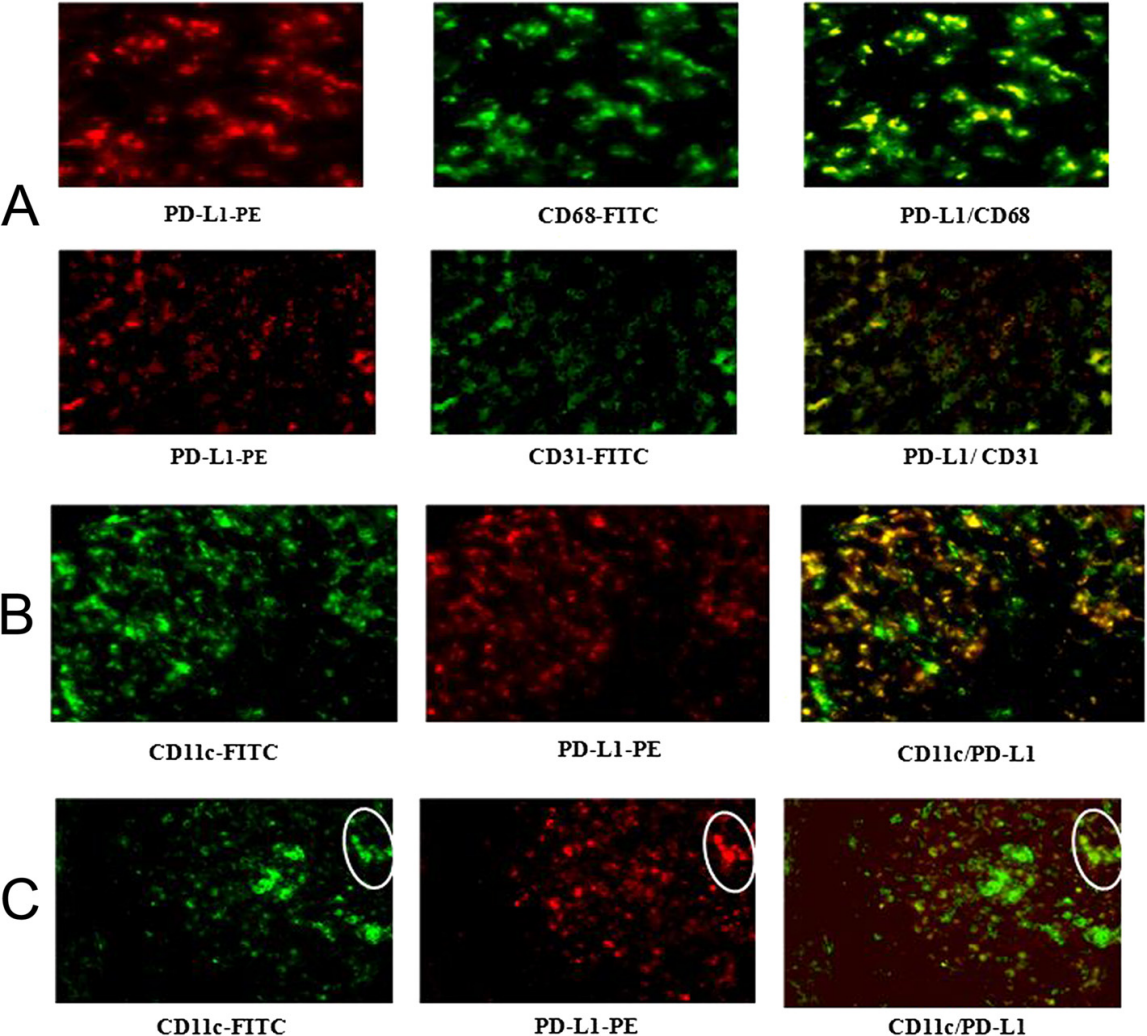
**(A) Co-localization of PD-L1 with CD31 and CD68 is shown by immunofluorescence double staining in liver biopsy specimens of patients with hepatitis B in T4.** PD-L1 (red) is co-localized with CD31 (green) positive endothelia cells or CD68 (green) positive Kupffer cells. The 2-color merged panels show co-localization visible (yellow). Original magnification × 200. (**B**) Co-localization of PD-L1 and CD11c in liver tissue from patients during T3. PD-L1 (red) is co-localized with CD11c (green). The 2-color merged panels show co-localization (yellow). Most CD11c + cells expressed PD-L1. Original magnification × 200. (**C**) Co-localization of PD-L1 and CD11c in liver tissue from patients in T4. PD-L1 (red) is co-localized with CD11c (green). The 2-color merged panels show co-localization (yellow). Most CD11c + cells did not express PD-L1. Only CD11c + cells in the circle expressed PD-L1. Original magnification × 200.

The number of intra-hepatic PD-L1 positive cells did not decrease significantly from T3 to T4 period. Immunofluorescence double staining of liver tissue from the T4 period showed that PD-L1 was primarily expressed on CD68-positive macrophages (Kupffer cells), and CD31-positive sinusoidal epithelial cells (Figure 7A). Intra-hepatic PD-L1 expression on CD11c + cells was also compared between T3 and T4 period. During T3, most CD11c + cells expressed PD-L1 (Figure 7B); while during T4, the majority of CD11c + cells did not (Figure 7C). Because the frequency of PD-L1 expression on circulating CD11c + cells decreased significantly from T3 to T4 period, there is good concordance between intra-hepatic and circulating PD-L1 expression on CD11c + cells.

### Dynamic analysis of TNF-α and IFN-γ mRNA expression from T1 to T4

**Figure 8:**
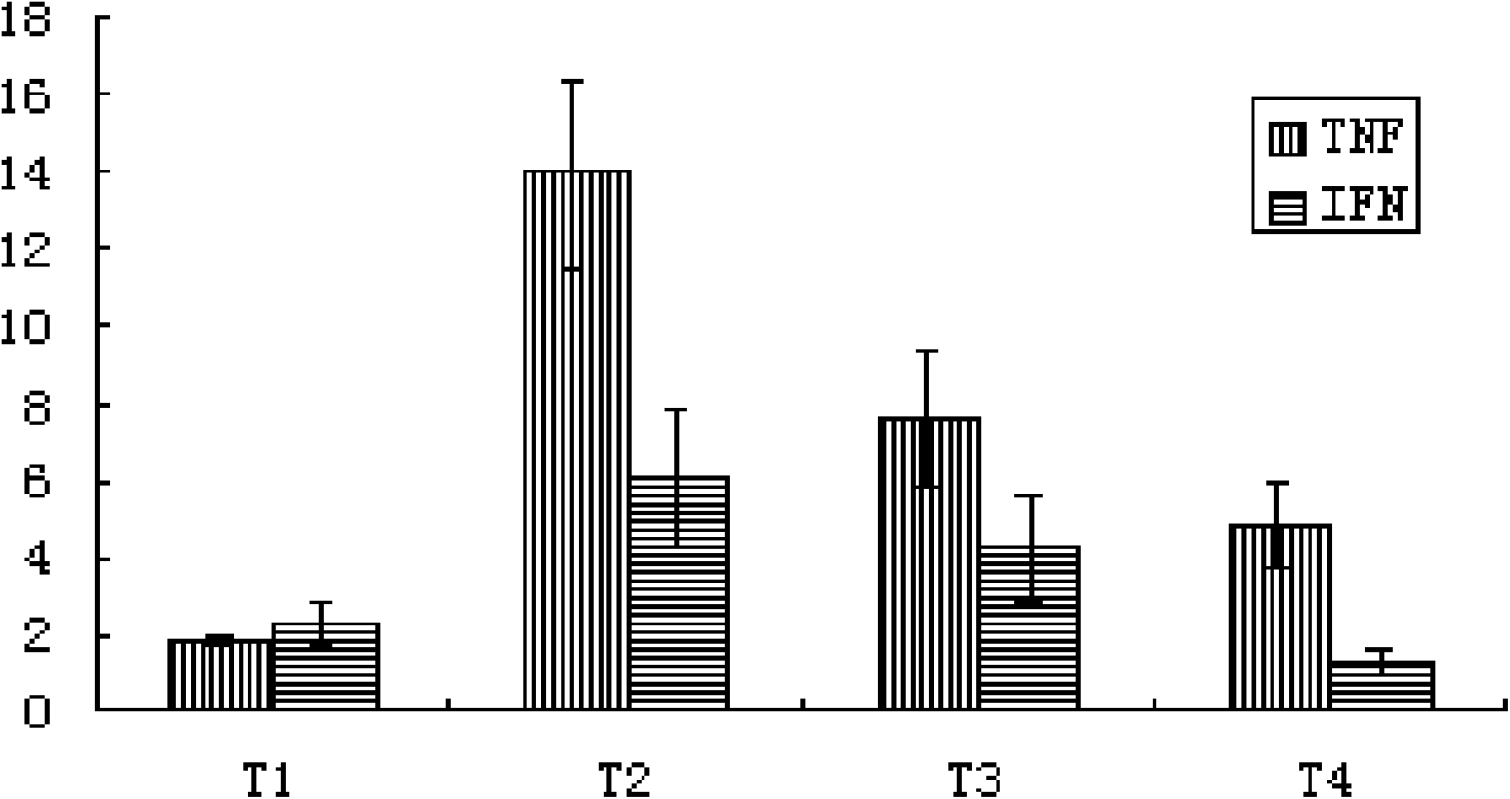
**Detection of TNF-α and IFN-γ mRNA expression by QT-PCR (n = 5).** The levels of TNF-α and IFN-γ mRNA expression significantly increased from T1 to T2, and decreased from T2 to T4. TNF-α expression level in T4 period was still significantly higher than that in T1. TNF-α mRNA was 2.5-fold higher in T4 than that in T1.

Pro-inflammatory cytokines expression levels were also dynamically detected by real-time PCR in chronic hepatitis B patients from T1 to T4 period. As shown in Figure 8, TNF-α and IFN-γ mRNA can be detected in patients in T1 period, and increased progressively, reaching peak levels during the climax phase of inflammation (T2) and decreasing rapidly during the decline phase (T3). During T4, IFN-γ mRNA levels decreased to less than those in the T1 period, while TNF-α mRNA expression were at a relatively high level. The TNF-α mRNA level further decreased from the T3 to the T4 period, but was still significantly higher than the levels found during T1 (Figure 8).

### Correlation between PD1/ PD-L_1_ expression and downstream inflammatory events

As shown in Figure 3, the circulating and intra-hepatic PD1/PD1-L expression were correlated with downstream inflammatory events, such as HBV-specific CD8 T cells expansion and contraction, TNFα and IFNγmRNA expression and changes in serum HBV-DNA and ALT levels.

The number of PD1 and PD-L1 positive cells, both intra- and extra-hepatic increased significantly during T2 compared to those in T1 period. In particular intra-hepatic PD1- and PD-L1-positive cells infiltrated extensively in the lobular and portal areas during T2. During the same phase, the number of pentamers such as core 18–27, pol 575–583 and even 335–343 also peaked rapidly, reaching levels greater than 4, 3 and 3.2 times higher than the levels in T1 respectively. Subsequently, the serum ALT level also increased to more than 10 times greater than the upper limit of normal paralleling marked decrease in HBV-DNA and HBsAg. During this period, TNFα and IFNγmRNA expression also reached peak levels.

During T3 period, the serum HBV-DNA levels progressively decreased to levels less than 10-fold below those found in T2, and serum HBsAg levels decreased greater than 2-fold. At the meantime, the number of pentamers, which include core18-27, even 335–343 and pol 575–583 cells also decreased significantly, paralleling the decrease of serum ALT, and intra-hepatic TNFα and IFNγmRNA expression levels. Concomitant with these changes, the levels of both the circulating and intra-hepatic PD1 and PD-L1 expression also decreased significantly.

During T4 period, serum HBV-DNA levels further decreased. The serum ALT also further declined, approaching normal level (less than 100u/L). Correlating very well with these changes, the frequency of circulating pentamers and the number of intra-hepatic CD8+ cells further decreased significantly and the intra-hepatic IFN-γ mRNA expression level decreased to less than that found in T1; however, TNFα mRNA maintained a relatively high level throughout the same period. The frequency of circulating PD1-positive cells decreased near to T1 levels, and almost all of the liver infiltrating PD1-positive cells aggregate in fibrous septa. It is noted that during this period, the number of intra-hepatic PD-L1-positive cells did not decrease significantly compared to that during the T3 period (Figure 3 A,B,C,D).

## Discussion

PD1, a negative regulator of the immune response, is predominantly expressed on activated T cells [10,11]. PD-L1, the ligand for PD1, is induced on APCs upon onset of activation [10,11]. The interaction between PD1 and PD-L1 transmits a co-inhibitory signal to downstream molecular events and then limits the activated T cell proliferation and cytotoxic effects, resulting in tissue protection against severe injury by an over-vigorous immune response [12-14]. During the inflammation flare up period in patients with chronic hepatitis B, the frequency of circulating PD1-positive cells and the number of intra-hepatic PD1-positive cells increased significantly and reached peak levels. During the same phase, circulating and intra-hepatic PD-L1 expression also increased significantly. Activated HBV specific T cells expressing PD1 were recruited to the liver and contacted PD-L1 expressing APCs (e.g. mDCs, kupffer cells, and LSECs). This interaction resulted in impaired functions of HBV specific-T cells. Kassel et al. reported that the upregulation of intra-hepatic PD-L1 may efficiently limit over-vigorous immune response, avoiding severe liver damage [14]. Chen et al. also reported that delayed PD1 up-regulation in patients with acute hepatitis B is closely associated with fulminant hepatitis[12].

Based on the Polly Matzinger’s viewpoint, the expression of co-inhibitory molecules is needed for the maintenance of homeostasis [15]. In chronic hepatitis B patients, during the immune-inactive phase, whose liver function was nearly normal, it is not necessary to increase significantly for circulating and intra-hepatic PD1 and PD-L1 expression. Our previous studies revealed that in chronic hepatitis B patients with immune-inactive state, PD-L1 positive cells were almost absent in liver lobe (data is not shown). Rachel Kassel et al. also reported that PD1 and PD-L1 expression always increased significantly in inflamed liver from chronic hepatitis B patients with immune-clearance state [14].

Although the up-regulation of PD1 and PD-L1 may benefit restoration of liver function and maintenance of homeostasis, it is also thought to facilitate replication of remaining HBV, finally resulting in persistent infection. In chronic hepatitis B patients, immune clearance phase and immune inactive phase always displayed alternatively. During the immune clearance phase, parallel with serum ALT elevation, serum HBV-DNA and HBsAg load declined, and may eventually lead to HBV-DNA seroclearance and HBsAg seroconversion to its antibody (anti-HBs) in part of patients; while following immune clearance phase, most of the patients enter an immune inactive phase with normal serum ALT, low serum HBV-DNA. In immune inactive phase, HBV-DNA may replicate actively again and HBV antigens may accumulated until it triggers a next round of immune response against HBV.

To elucidate the role of PD1 and PD-L1 in the mechanism of these two distinct states alternation, PD1 and PD-L1 expression were analyzed longitudinally in present study throughout the immune clearance phase; and the changes of PD1 and PD-L1 expression were related to HBV -specific T-cell clonal expansion, proinflammatory cytokine expression, serum ALT, HBV-DNA and HBs-Ag fluctuation.

During the T2 phase, HBV specific T cells, including core 18–27, even353-341 and pol 575–583, expanded rapidly reaching the levels more than 35, 30, 37 times higher than healthy control level (which never exceeded 0.08%), respectively. Concomitant with HBV- specific T-cell clonal expansion, serum ALT reached the peak levels, followed by a significant decreased in serum HBV-DNA and HBsAg load. During this period, the percentage of PD1 expression of total CD8 cells increased from 13.1% ± 4.2% in T1 phase to 38.7% ± 8.3% in T2 phase. The frequency of PD1 expression on pentamers was greater than 50% in the T2 phase.

During the T3 phase, the frequency of HBV-specific CD8 + T-cells decreased significantly, serum ALT level decreased by more than half of that in the T2 period, followed by further viremia reduction. During this period, the frequency of circulating PD1 expression further decreased significantly.

In T4, serum HBV-DNA further decreased to levels lower than those found in T3 and serum ALT level decreased to less than 100 IU/L. The frequency of HBV-specific CD8 + T-cells further decreased to less than 1.0% and the frequency of circulating PD1 expression also decreased to less than the level found in T1.

Intra-hepatic PD1 expression was detected throughout the clinical course from T2 to T4. In the T2 phase, CD8+ and PD1+ cells infiltrated extensively in lobular and portal areas. The number of infiltrating PD1-positive cells decreased significantly from 388 ± 122/hpf in T2 to 174 ± 53/hpf in T3. In the T4, the number of intrahepatic PD1+ cells further decreased significantly, and most PD1-positive cells could be observed aggregated in fibrous septa and in periportal regions. Our previous findings showed that almost all liver-infiltrating CD8 T-cells expressed the home receptor CCR5 and CCR7 (data is not shown). This phenomenon was consistent with previous results that PD1-positive CD8 T-cells home from peripheral circulation to lobular and portal areas to form portal tract- associated lymphoid tissue. [16-18].

Two conclusions can be drawn based on these data. First, CD8 T cells maintain normal proliferative capacity towards HBV antigens and undergo colon expansion during inflammatory episodes in patients with chronic hepatitis B. Second, PD1 expression is located primarily on activated HBV specific T cells and changes are associated with HBV-specific T-cell clonal expansion and contraction, serum ALT and viral load changes. These data are in line with previous findings which showed that PD1 expression is induced and up-regulated significantly on activated HBV-specific CD8 T-cells during colon expansion period [8,12,14].

PD-L1 expression exhibits a similar dynamic pattern in patients with chronic hepatitis B. The expression of PD-L1 on circulating CD11c + mDCs increased during the initial phase of liver inflammation (T1) and reached peak levels in parallel with up-regulation of the frequency of PD1 expression on circulating CD8+ T cells in the T2 period. In the T3 period, following a decline in circulating PD1 expression, the frequency of PD-L1 expression also decreased significantly. In the regression phase (T4), associated with the decline of PD1 expression, the frequency of circulating PD-L1 decreased further, approaching to the levels in T1 period. These data indicate that there is good concordance between circulating PD-1 and PD-L1; it changes during the inflammation flare period.

Intra-hepatic PD-L1 expression was detected from T2 to T4 phases of chronic hepatitis B. During the climax phase of inflammation (T2), intra-hepatic PD-L1 positive cells were observed extensively in lobular areas. The number of these cells decreased significantly in the decline phase of inflammation (T3) compared to the levels in T2. It did not decrease significantly from T3 to T4 period although the frequency of circulating PD-L1 decreased significantly from 28.86% ± 4.95% to 11.18% ± 3.54% in the same period. Note, in T3 period, most of liver infiltrating CD11c + cells were PD-L1 positive, while in T4, intrahepatic PD-L1 positive cells were CD68+ Kupffer and CD31+ endothelial cells, and the majority of intrahepatic CD11c were PD-L1 negative.

Studies have reported that PD-L1 expression can be induced by pro-inflammatory cytokines such as IFNγand TNFα in an antigen-independent manner [19-22]. During the regression period (T4), the mRNA expression level of TNFα was maintained at a higher level compared to that during the T1 period. This result suggests that in liver tissue, a relatively high level of TNFα expression may attribute to intrahepatic PD-L1 up-regulation in the T4 phase.

In conclusion, these results suggest that persistent up-regulation of intra-hepatic PD-L1 on CD68 kupffer and CD31 endothelia may constitute an immunosuppressive microenvironment during the regression phase (T4). The effects of PD1 expressing HBV-specific T-cells are dampened when they home the liver and come in contact with PD-L1-expressing CD68+ Kupffer and CD31+ endothelial cells. This may facilitate replication of remaining HBV-DNA and finally lead to recurrence of hepatitis.

## Conclusion

In general, the levels of co-inhibitory molecules expression were increasing progressively in parallel with ascending inflammation and were decreasing significantly during inflammatory regression phase. Contrary to this phenomenon, intra-hepatic PD-L1 expression on non-parenchyma cells did not decrease significantly in inflammatory regression phase. This may contribute to constitute immunosuppressive microenvironment which may damp HBV-specific immune response and finally lead to persistent HBV infection.

## Competing interest

The authors declare that they have no competing interests.

## Authors’ contributions

Zhang Wenjin contributed to study concept, study design, writing of the manuscript. Zheng Shusen contributed to study concept and study design. Peng Chuanhui contributed to data acquisition, statistical analysis. Wan Yunle contributed to data acquisition, statistical analysis. Shaikh Abdul Lateef contributed to writing of the manuscript. All authors read and approved the final manuscript.

Support by: Zhejiang Provincial Natural Science Foundation, No. Y2110169.

## Pre-publication history

The pre-publication history for this paper can be accessed here:

http://www.biomedcentral.com/1471-230X/12/109/prepub
